# Seed Composition and Amino Acid Profiles for Quinoa Grown in Washington State

**DOI:** 10.3389/fnut.2020.00126

**Published:** 2020-08-12

**Authors:** Evan B. Craine, Kevin M. Murphy

**Affiliations:** Department of Crop and Soil Sciences, Washington State University, Pullman, WA, United States

**Keywords:** quinoa, essential amino acid, limiting, complete protein, protein quality

## Abstract

Quinoa (*Chenopodium quinoa* Willd.) is a pseudocereal celebrated for its excellent nutritional quality and potential to improve global food security, especially in marginal environments. However, minimal information is available on how genotype influences seed composition, and thus, nutritional quality. This study aimed to provide a baseline for nutritional quality of Washington grown quinoa and test the hypothesis that these samples contain adequate amounts of essential amino acids to meet daily requirements set by the World Health Organization (WHO). One hundred samples, representing commercial varieties and advanced breeding lines adapted to Washington State, were analyzed for content of 23 amino acids, as well as crude protein, ash, moisture, and crude fat. Mean essential amino acid values for Washington grown quinoa met the daily requirements for all age groups for all essential amino acids, except for the amount of leucine required by infants. We found that only nine genotypes met the leucine requirements for all age groups. A total of 52 and 94 samples met the lysine and tryptophan requirements for all age groups, respectively. Mean values for isoleucine, leucine, lysine, tryptophan, valine, and the sulfur and aromatic amino acids are higher for Washington grown samples than those reported previously reported in the literature. Our results show that not all Washington grown quinoa samples meet daily requirements of essential amino acids, and we identify limiting amino acids for the germplasm and environments investigated. This study provides the first report of leucine as a limiting amino acid in quinoa. Additional research is needed to better understand variation in quinoa nutritional composition, identify varieties that meet daily requirements, and explore how genotype, environment, and management interactions influence nutritional quality.

## Introduction

Andean farmers have domesticated, adapted, diversified, and conserved quinoa genetic resources for the last 7,000 years, and until recently quinoa has been regarded as a neglected and underutilized species (NUS) (1, 2). Quinoa is a gynomonecious allotetraploid and a facultative autogamous annual species in the Amaranthaceae family, with a base chromosome number of x = 9 (2n = 4x = 36) and outcrossing from 0.5 to 17.36% (2–5). Due to domestication along latitudinal and elevational gradients, quinoa exhibits a large amount of genetic diversity and adaptive capacity (6). Two germplasm pools exist representing major centers of diversity, one in the Andean highlands, and the other in central and southern Chilean coastal lowlands (2, 7, 8). Quinoa is grown in a wide range of environments, is resilient to agro-ecological extremes, and is tolerant to several abiotic stressors (e.g., drought, salinity, frost) (9–11). The broad genetic variability and adaptability of quinoa to diverse climates has produced a gene pool that supports the strategic development of germplasm with varying morphological (12) and physiological (13, 14) characteristics, and end-uses (15–18) suitable for adoption in novel agroecological climates worldwide. However, the germplasm pool currently available to facilitate quinoa expansion and adoption in novel production regions is narrow and represents only a small portion of quinoa's genetic diversity. The germplasm pool is primarily constrained by physiological issues (e.g., grain filling) associated with day length sensitivity (19).

Quinoa has the potential to improve the functional properties and nutritional quality of a diverse range of dishes and food products, from traditional and non-traditional applications to industrial innovations. These include soups, desserts, pastries, hot and fermented drinks (traditional), cereals, granolas, snack bars, cakes, chocolates (non-traditional), and extruded, puffed and expanded products (industrial innovations). Quinoa can also be used to produce almost all products of the milling industry (20–24). Moreover, quinoa flour has a major advantage in the flour industry since it can meet the increasing international demand for gluten free products (25–27). Quinoa protein, oil, and starch fractions can be isolated for specific value-added applications. Quinoa has good freeze-thaw capabilities and the gelatinized starch is opaque, which makes quinoa useful in prepared frozen foods and as an emulsifier, respectively (28, 29). The wide range of quinoa starch physicochemical properties provides for diverse applications in food and non-food innovations (16, 30, 31).

Quinoa starch is more viscous and has better water holding and expansion properties than wheat and barley, and starch gelatinization occurs at higher temperatures, making quinoa perform better as a thickening agent and in baby foods (12, 26). López de Romaña and others have used quinoa in two studies with Peruvian children recovering from malnutrition. In both studies, a lower digestibility of quinoa was observed compared to potato and wheat diets and casein, which contributed to decreased protein and fat utilization; however, milling improved both parameters (32, 33). Processing methods and quinoa variety both contribute to variability in end-use quality (16, 30). Additional research is needed to characterize nutritional quality and functional properties depending on variety and processing, to ensure successful utilization of quinoa in appropriate end-uses.

The superior nutritive potential of quinoa has relatively recently garnered international interest in the expansion of quinoa (19). It is beyond the scope of this article to provide a comprehensive review of quinoa nutritional composition, since several already exist (34–39). Quinoa protein content can be highly variable, and is often comparable to most cereals, ranging from 8 to 22% (37, 40). The quality of quinoa protein is noteworthy. Quinoa is reported to be a complete protein, because it contains all nine of the essential amino acids (35, 37, 41); however, it is better described as “nearly complete,” because of limiting essential amino acid content. In a review of quinoa data reported as edible portion, which allows for comparison to food composition databases, Nowak et al. (37) had to relax the data selection criteria for amino acids and minerals because of a lack of information. Although the authors claimed that quinoa provides a sufficient amount of essential amino acids, even at the lower ends of the ranges to meet adult daily requirements, this is based on a miniscule number of data points (*n* = 37) (37). Furthermore, the authors neglected to evaluate the ability of quinoa to meet the daily essential amino acid requirements of younger age groups. Far too much has been inferred about quinoa nutrition composition from the limited number of peer-reviewed studies available.

By declaring 2013 as the “International Year of Quinoa,” the United Nations recognized the emerging potential of quinoa to contribute to global food security, especially when grown on marginal lands that cannot currently support major crops (1). It has been reported that quinoa has been tested or cultivated in 95 countries, a doubling since the declaration in 2013, and it appears that this trend will continue (19, 42). However, the large amount of genetic diversity, resiliency to agroecological extremes, and diversity of morphological and physiological characteristics is not necessarily represented in the germplasm pool currently supporting the global expansion of quinoa (42, 43). Furthermore, and perhaps most importantly, the nutritional quality of quinoa produced in novel environments is assumed to be comparable to the quality of quinoa produced in Bolivia and Peru, which represents roughly 80% of global production (19). Thus, this study aims to provide baseline information on quinoa grown in western North America (i.e., Washington state), representing the first report that provides a baseline for the protein quality of quinoa produced in this novel production region. We also test the hypothesis that Washington grown quinoa contains adequate amounts of essential amino acids to meet daily requirements set by the World Health Organization (WHO) for all age groups. Comparisons are made to not only adult requirements, but also to the requirements for infants and all other age groups. It is estimated that between 25 and 33% of children below the age of five experience stunting worldwide, possibly due to insufficient protein intake (44, 45). For example, Semba et al. (46) found that 62% of the children in their study in rural Malawi were stunted, and that these children had lower serum concentrations of all nine essential amino acids compared to non-stunted children (*p* < 0.01). Furthermore, the stunted children had significantly lower serum concentrations of conditionally and non-essential amino acids (46). Therefore, providing adequate nutrition to children under the age of five, especially regarding sufficient intake of amino acids, is critically important for reducing the risk of stunting and effects on growth and development (47, 48).

## Methods

### Study Region and Field Trials

Raw quinoa seed sent for analysis was grown in 2016 and 2017 in western Washington as part of two separate experimental designs (13, 49). Site characteristics for all locations are summarized in Table 1. In 2016, F5:F6 advanced breeding lines and control varieties were planted on three organic farms in Chimacum (Finnriver Farm; 48°0'29“N 122°46'12”W), Quilcene (Dharma Ridge Organic Farm; 47°55'04.0“N 122°53'23.2”W) and Sequim (Nash's Organic Produce; 48°08'31“N 123°07'19”W) on the Olympic Peninsula. Control varieties included Cherry Vanilla (Wild Garden Seed, Philomath, OR, US), CO407Dave (PI 596293, USDA Plant Introduction, Ames, Iowa) and Kaslaea (Ames 13745, USDA Plant Introduction, Ames, Iowa). At each location, advanced breeding lines and control varieties were planted in single hand-sown plots that measured 4.9 m in length and 40.64 cm from center and were seeded at a rate of 4 g row m^−1^ in an augmented randomized complete block design (ARCBD). An ARCBD uses control varieties to account for field variation by replicating control varieties across blocks; control varieties can be used as covariates to make spatial adjustments across blocks. This design is useful for evaluating advanced breeding lines when seed quantity is low, land and other resources are limited, and when many advanced breeding lines must be evaluated.

**Table 1:** Site characteristics for each year and location that samples were randomly selected from for chemical analysis. Raw quinoa seed sent for analysis was grown in 2016 and 2017 in western Washington as part of two separate experimental designs (13, 49).

	**2016**			**2017**
	**Chimacum**	**Quilcene**	**Sequim**	**Mount Vernon**
Elevation (m)	37.8	68	31.1	7
Average Annual Precipitation (mm)	711	1397	432	841
Planting Date	April 8, 2016	April 7, 2016	May 5, 2016	May 18, 2017
Soil Type	Gravelly sandy loam	Silty Clay Loam	Silt Loam	Silt Loam
pH	6.1	6.9	6.5	6.6
Phosphorus (mg kg^−1^)	124	12	31	7
Potassium (mg kg^−1^)	762	290	114	260
Ammonium (mg kg^−1^)	1.1	5.7	4.5	1.2
Nitrate (mg kg^−1^)	21.5	16.6	3.8	25^†^
Organic Matter (%)	12.1	3.2	3.3	2.9
Previous Crop	Barley	Vegetable Crops	Pasture	Rye Cover Crop

*Mount Vernon plants were planted in the greenhouse 1 month before being transplanted on the planting date listed*.

† *Reported as nitrate + nitrite*.

In 2017, quinoa seeds were planted in a greenhouse, and after 1 month the seedlings were transplanted in the field on 19 May at the WSU Northwest Research and Extension Center in Mount Vernon, WA (NWREC; 48°26'24“N 122°23'24” W). The experimental design in 2017 consisted of a split-plot randomized complete block design with irrigation factor (irrigated, non-irrigated) as the main-plot and genotype as the sub-plot. Each plot had a distance of 30 cm between plants, with 30 plants in each plot. Following harvest each year, seed was dried at 32°C and cleaned using metal screens and a seed blower (Seed Processing Holland Inc., Salinas, CA).

### Germplasm and Sample Selection

The F5:F6 advanced breeding lines were generated from single plant selections made on six bi-parental populations through an evolutionary participatory breeding (EPB) method (49). Crossing events in 2012 produced the six, original bi-parental populations (50) (Table 2). Germplasm included in the 2017 trial represents commercially available varieties and landraces. A summary of the germplasm is included in Table 3.

**Table 2:** Female and pollen parents listed for each bi-parental population, with the number of samples from each population included in the study.

**Population**	**Female parent**	**Pollen parent**	**Number of samples**
102	CO407 Dave	QQ74	21
104	Kaslaea	QQ74	26
105	QQ065	QQ74	3
106	QQ065	Black	5
107	QQ74	Black	10
108	QQ74	Cherry Vanilla	20

**Table 3:** Summary of samples from both years, each location, and the identity of the advanced breeding lines (ABL) and control varieties (CV).

**2016 (*****n*** **=** **91)**						**2017 (*n* = 9)**
**Ch (*****n*** **=** **24)**		**Qu (*****n*** **=** **37)**		**Sq (*****n*** **=** **30)**		**MV (*n* = 9)**
**ABL**	**CV**	**ABL**	**ABL**	**ABL**	**CV**	**Accessions**
102.04	Cherry Vanilla•	102.05	107.03	102.08•	Cherry Vanilla•	17GR (Ames 13735)^††^
102.08•		102.09	107.07	102.17•		Japanese Strain (PI 677100)^††^
102.13	CO407 Dave•	102.12	107.50	102.24	CO407 Dave•	QQ74 (PI 614886)^††^
102.31	Kaslaea•	102.17•	107.65•	102.36•	Kaslaea•	Baer (PI 634918)^†^
102.52•		102.23	107.72	102.52•		3 UISE (Ames 13756)^††^
102.76••		102.25	107.84	102.76••		
104.01		102.36•	108.18	104.20••		
104.02		102.40	108.51•	104.21•		
104.20••		102.76••	108.56	104.27•		
104.27•		104.20••	108.66	104.28•		
104.60		104.21•	108.70	104.45		
104.73•		104.28•	108.86•	104.59•		
105.43		104.30	108.90	104.71		
107.67		104.38		104.80		
107.78•		104.53		104.88		
108.33		104.59•		105.92•		
108.34		104.73•		106.37•		
108.39		104.75		106.49•		
108.46		104.77		107.65•		
108.51•		104.87		107.78•		
108.54•		105.92•		108.11		
		106.37•		108.26		
		106.49•		108.42		
		106.85		108.54•		
				108.69		
				108.81		
				108.86•		

*ABL are denoted by the population number and selection number separated by a period. In 2016, ABL and CV were grown at Chimacum (Ch), Quilcene (Qu), and Sequim (Sq). Samples were randomly selected from the 2016 experimental design; certain ABL were selected from two (•) or three (••) locations and none of the CV were selected from Quilcene by chance. Samples were also randomly selected from the Mount Vernon (MV) experimental design, from either the non-irrigated treatment (†) or both the non-irrigated and irrigated treatments (††)*.

Protein values were predicted for a representative selection of field-grown material (*n* = 194), including samples from the two aforementioned experimental designs, using a DA7250 NIR analyzer (Perten Instruments, Springfield, IL) with a default quinoa calibration. Predicted values were normally distributed, and samples included in the study were randomly selected across the distribution for wet chemistry analyses (*n* = 100), with a greater number of samples selected within one standard deviation of the mean to better represent this dense region of the distribution.

### Chemical Laboratory Analysis

Samples were sent to the (AESCL) for determination of seed composition via proximate analysis (crude protein, crude fat, moisture, ash, and carbohydrates) and determination of the complete amino acid profiles (*n* = 23) [AOAC Official Method 982.30 E(a,b,c), chp. 45.3.05]. AESCL is an American Association for Laboratory Accreditation (A2LA) accredited proficiency testing provider in accordance with the international standard 17043:2010. This accreditation demonstrates technical competence for a defined scope and the operation of a quality management system.

The complete amino acid profile included essential amino acids (leucine, lysine, valine, isoleucine, phenylalanine, threonine, histidine, methionine, and tryptophan) and non-essential amino acids (glutamic acid, aspartic acid, arginine, glycine, alanine, proline, serine, tyrosine, cysteine, taurine, hydroxyproline, hydroxylysine, ornithine, and lanthionine). Crude protein was determined by combustion analysis (LECO), and the calculation of total nitrogen × 6.25 [AOAC Official Method 990.03 (51)]. Crude fat was determined by ether extraction [AOAC Official Method 920.39 (A)]. Moisture was determined by vacuum oven [AOAC Official Method 934.01 (51)] and ash was determined by sample ignition (AOAC Official Method 942.05). Total carbohydrates were determined by difference calculation [100—(Crude Protein + Crude Fat + Ash + Moisture)]. Proximate values are reported as g/100 g sample, and amino acids are reported as g/100 g crude protein, unless otherwise noted.

### Daily Requirements and Scoring Patterns

FAO/WHO/UNU (52) scoring patterns should be based on amino acid requirement values divided by the mean protein requirement, and are presented as g/100 g protein (Table 4). Scoring patterns are calculated as the age-related amino acid requirement levels divided by the safe level of protein intake (52).

**Table 4:** Daily requirements for essential amino acids presented as scoring patterns (amino acid requirements/protein requirements for the selected age groups) for all age groups considered.

**Scoring pattern (g/100 g protein requirement)**									
**Age (years)**	**His**	**Ile**	**Leu**	**Lys**	**SAA**	**AAA**	**Thr**	**Trp**	**Val**
0.5	2	3.2	6.6	5.7	2.8	5.2	3.1	0.85	4.3
1–2	1.8	3.1	6.3	5.2	2.6	4.6	2.7	0.74	4.2
3–10	1.6	3.1	6.1	4.8	2.4	4.1	2.5	0.66	4
11–14	1.6	3	6	4.8	2.3	4.1	2.5	0.65	4
15–18	1.6	3	6	4.7	2.3	4	2.4	0.63	4
>18	1.5	3	5.9	4.5	2.2	3.8	2.3	0.6	3.9

*Amino acids are abbreviated with standard three letter codes. The sulfur amino acids (SAA) include methionine and cysteine and the aromatic amino acids (AAA) include phenylalanine and tyrosine. This table is adapted from the World Health Organization/Food and Agriculture Organization/United Nations University suggested indispensable amino acid requirements for all age groups (present estimates; 2007)*.

### Data Analysis

Data were analyzed and figures were generated using Microsoft Excel 2010 (Seattle, WA), and RStudio Version 1.2.1335 (53). Data was tested for normality using the *shapiro.test* function in the *stats* R package. An overall rank score was assigned to each sample by summing individual ranks for each nutritional attribute (Table 4). For example, the samples with the highest and lowest essential amino acid content received a rank of 1 and 100, respectively. Spearman rank correlation coefficients were calculated using the *corstars* function and *Hmisc* R package, with significant correlations (*p* < 0.05) plotted using the *corrplot* package and diverging palette “RdBu” from the *RColorBrewer* package (54–56). Principal component analysis was conducted using the *prcomp* function in the *stats* package, and biplots were generated using the *ggplot2* package (53, 57). The *tabular* function in the *tables* package provided summary statistics (58).

## Results

### Seed Composition (Proximates) Profile

Quinoa grown in Chimacum (*n* = 24; advanced breeding lines and control varieties; Table 3) had the highest mean total amino acid, crude protein and moisture content, and the lowest mean crude fat content (Table 5). Quinoa grown in Mount Vernon (*n* = 9; accessions; Table 3) had the highest mean ash content, and the lowest mean total amino acid, crude protein, moisture, and total carbohydrate content. Quinoa grown in Quilcene (*n* = 37; advanced breeding lines; Table 3) had the highest mean total carbohydrate content, whereas quinoa grown in Sequim had the highest mean crude fat content and the lowest mean ash content (Table 5). Our sample of Washington grown quinoa seeds is primarily composed of total carbohydrates (69.56–74.00 g/100 g sample) followed by crude protein (10.04–13.68 g/100 g sample), moisture (6.41–7.37 g/100 g sample), crude fat (4.56–7.19 g/100 g sample), and ash (2.70–5.00 g/100 g sample) (Table 5).

**Table 5:** Mean and standard deviation (sd) values for the nutritional components (NC) analyzed are reported for all samples (*n* = 100), and the samples grouped by location and population (i.e., seed source).

			**Location**				**Population**						
		**All**	**Ch**	**MV**	**Qu**	**Sq**	**CV**	**102**	**104**	**105**	**106**	**107**	**108**
**NC**	*n*	100	24	9	37	30	6	21	26	3	5	10	20
Carb	mean	72.27	71.83	72.07	72.74	72.09	71.68	72.55	72.48	72.17	71.85	71.90	72.27
	sd	0.87	0.84	0.89	0.73	0.80	0.80	0.64	0.88	0.69	1.74	0.74	0.80
CP	mean	11.77	12.25	11.26	11.46	11.91	12.09	11.70	11.75	12.23	12.01	11.84	11.84
	sd	0.70	0.73	0.87	0.54	0.50	0.81	0.55	0.72	0.70	0.85	0.71	0.63
Moist	mean	6.97	7.08	6.60	7.03	6.91	7.04	6.96	7.01	6.99	7.11	7.04	6.98
	sd	0.20	0.08	0.17	0.13	0.20	0.09	0.16	0.16	0.29	0.15	0.19	0.16
Fat	mean	5.89	5.73	5.82	5.79	6.16	6.19	5.89	5.79	5.56	5.87	6.17	5.88
	sd	0.50	0.35	0.72	0.47	0.48	0.59	0.34	0.46	0.23	0.96	0.70	0.26
Ash	mean	3.11	3.12	4.25	2.97	2.93	3.00	2.90	2.98	3.05	3.15	3.06	3.04
	sd	0.41	0.18	0.35	0.17	0.12	0.14	0.08	0.16	0.10	0.29	0.26	0.16
TAA	mean	87.11	88.41	84.49	87.71	86.10	89.09	87.28	86.61	89.47	88.52	86.83	87.60
	sd	3.61	3.87	3.48	3.16	3.46	4.92	4.67	2.89	1.01	2.37	3.20	3.15
TEAA	mean	34.13	34.53	32.79	34.47	33.78	34.92	34.37	33.97	34.68	34.50	33.93	34.36
	sd	1.42	1.56	1.11	1.27	1.29	1.93	1.84	1.13	0.15	0.95	1.37	1.17
His	mean	2.66	2.72	2.53	2.65	2.66	2.74	2.65	2.64	2.75	2.79	2.65	2.68
	sd	0.14	0.15	0.12	0.12	0.13	0.15	0.16	0.11	0.04	0.06	0.12	0.12
Ile	mean	4.00	4.09	3.90	4.00	3.96	4.07	4.00	3.95	4.14	4.16	4.03	4.01
	sd	0.18	0.19	0.16	0.16	0.16	0.22	0.23	0.14	0.08	0.05	0.18	0.16
Leu	mean	6.25	6.35	6.04	6.31	6.16	6.38	6.28	6.18	6.41	6.41	6.27	6.28
	sd	0.26	0.27	0.21	0.24	0.23	0.33	0.34	0.21	0.05	0.21	0.25	0.21
Lys	mean	5.72	5.64	5.33	5.92	5.65	5.82	5.84	5.76	5.62	5.65	5.60	5.79
	sd	0.33	0.32	0.27	0.26	0.26	0.37	0.38	0.25	0.12	0.32	0.31	0.28
SAA	mean	4.02	4.05	3.87	4.06	4.00	4.17	3.97	4.05	4.12	3.95	3.94	4.09
	sd	0.19	0.18	0.23	0.17	0.19	0.23	0.23	0.15	0.26	0.09	0.10	0.12
Met	mean	2.17	2.20	1.97	2.21	2.14	2.26	2.15	2.20	2.24	2.15	2.12	2.22
	sd	0.13	0.11	0.15	0.11	0.10	0.17	0.12	0.10	0.15	0.07	0.10	0.08
AAA	mean	6.65	6.74	6.55	6.70	6.54	6.81	6.67	6.56	6.81	6.65	6.66	6.68
	sd	0.28	0.29	0.28	0.26	0.27	0.39	0.36	0.21	0.12	0.33	0.30	0.23
Phe	mean	3.90	3.96	3.82	3.94	3.84	3.99	3.92	3.86	4.03	3.98	3.92	3.92
	sd	0.16	0.18	0.14	0.15	0.15	0.21	0.21	0.13	0.05	0.14	0.16	0.14
Thr	mean	3.57	3.58	3.47	3.64	3.50	3.62	3.63	3.55	3.57	3.55	3.53	3.58
	sd	0.16	0.16	0.13	0.15	0.15	0.19	0.22	0.14	0.06	0.18	0.13	0.13
Trp	mean	1.08	1.14	1.00	1.01	1.15	1.15	1.12	1.11	1.04	0.93	1.03	1.09
	sd	0.14	0.11	0.07	0.16	0.10	0.09	0.16	0.14	0.03	0.22	0.10	0.12
Val	mean	4.78	4.85	4.73	4.79	4.72	4.89	4.79	4.72	4.88	4.87	4.79	4.81
	sd	0.20	0.22	0.18	0.19	0.20	0.26	0.27	0.17	0.05	0.14	0.22	0.16
TNAA	mean	52.98	53.88	51.70	53.24	52.32	54.16	52.91	52.64	54.79	54.02	52.90	53.24
	sd	2.26	2.33	2.57	1.94	2.22	3.03	2.87	1.79	0.86	1.44	1.87	2.04
Glu	mean	13.22	13.50	13.02	13.08	13.23	13.46	13.14	13.11	13.81	13.77	13.22	13.24
	sd	0.78	0.68	1.07	0.71	0.82	0.99	0.91	0.62	0.36	0.42	0.66	0.78
Asp	mean	8.00	8.09	7.80	8.12	7.84	8.14	8.01	7.93	8.32	8.32	8.07	7.97
	sd	0.35	0.35	0.29	0.30	0.34	0.43	0.45	0.30	0.06	0.22	0.32	0.27
Arg	mean	7.64	7.80	7.23	7.65	7.64	7.93	7.54	7.64	8.04	7.97	7.62	7.72
	sd	0.43	0.45	0.63	0.35	0.39	0.45	0.46	0.35	0.17	0.24	0.35	0.36
Gly	mean	5.54	5.49	5.47	5.63	5.49	5.63	5.56	5.51	5.61	5.63	5.48	5.57
	sd	0.24	0.27	0.33	0.19	0.23	0.35	0.29	0.21	0.05	0.22	0.22	0.21
Ala	mean	4.30	4.35	4.12	4.37	4.22	4.38	4.36	4.28	4.33	4.29	4.28	4.32
	sd	0.20	0.19	0.16	0.18	0.17	0.23	0.26	0.16	0.03	0.20	0.19	0.15
Pro	mean	3.85	4.04	3.69	3.85	3.75	3.96	3.80	3.82	4.21	3.90	3.89	3.91
	sd	0.24	0.21	0.13	0.21	0.22	0.28	0.26	0.22	0.25	0.22	0.22	0.22
Ser	mean	3.76	3.74	3.68	3.83	3.69	3.77	3.80	3.74	3.85	3.85	3.77	3.71
	sd	0.17	0.18	0.18	0.15	0.17	0.19	0.21	0.17	0.12	0.25	0.14	0.13
Tyr	mean	2.74	2.78	2.72	2.75	2.70	2.82	2.76	2.71	2.78	2.67	2.75	2.76
	sd	0.13	0.13	0.15	0.12	0.14	0.18	0.15	0.10	0.07	0.21	0.14	0.11
Cys	mean	1.85	1.84	1.89	1.85	1.85	1.92	1.82	1.85	1.89	1.80	1.82	1.87
	sd	0.09	0.09	0.13	0.08	0.10	0.07	0.12	0.08	0.11	0.07	0.03	0.06
Tau	mean	1.39	1.54	1.14	1.48	1.25	1.44	1.45	1.44	1.36	1.23	1.33	1.45
	sd	0.19	0.16	0.12	0.13	0.11	0.25	0.16	0.14	0.17	0.24	0.19	0.18
Hpro	mean	0.47	0.45	0.62	0.47	0.45	0.51	0.45	0.43	0.39	0.45	0.48	0.49
	sd	0.10	0.09	0.11	0.08	0.09	0.10	0.07	0.08	0.14	0.07	0.09	0.08
Hlys	mean	0.13	0.17	0.22	0.08	0.12	0.12	0.13	0.11	0.13	0.08	0.12	0.13
	sd	0.06	0.05	0.04	0.02	0.05	0.06	0.06	0.04	0.05	0.06	0.04	0.07
Orn	mean	0.09	0.08	0.10	0.09	0.09	0.08	0.09	0.09	0.08	0.08	0.08	0.09
	sd	0.01	0.01	0.03	0.01	0.02	0.01	0.01	0.01	0.00	0.01	0.01	0.02
Lan	mean	0.00	0.00	0.00	0.00	0.00	0.00	0.00	0.00	0.00	0.00	0.00	0.00
	sd	0.00	0.00	0.00	0.00	0.00	0.00	0.00	0.00	0.00	0.00	0.00	0.00

*The control varieties (CV) included were only selected from the Chimacum (Ch) and Sequim (Sq) locations by chance, while advanced breeding lines from the populations (102, 104, 105, 106, 107, and 108) were randomly selected from Ch, Sq, and Quilcene (Qu). Data from the Mount Vernon samples (n = 9) are only included under the “MV” location and “All” column. The first five NC, total carbohydrates (Carb), crude protein (CP), moisture (Moist), crude fat (Fat), and ash are reported as grams per 100 g sample. All amino acids are abbreviated using standard letter codes, except for total amino acid content (TAA), total essential amino acid content (TEAA) sulfur amino acids (SAA; methionine and cysteine), aromatic amino acids (AAA; phenylalanine and tyrosine); total non-essential amino acids (TNAA), taurine (Tau), hydroxyproline (Hpro), hydroxylysine (Hlys), ornithine (Orn), and lanthionine (Lan). All amino acid data are reported as grams per 100 g protein*.

Control varieties grown in Chimacum and Quilcene in 2016 (i.e., CTRL seed source) had the highest mean total amino acid and crude protein content (Table 5). Population 102 had the highest mean ash content. Samples from Population 105 had the highest total carbohydrate content and the lowest total amino acid, crude protein, and moisture. Population 106 had the lowest ash content. Population 107 had the lowest mean crude fat content. Samples from Population 108 had the highest mean crude fat content, and the lowest total carbohydrate content.

### Amino Acid Profile

The most abundant essential amino acids (*n* = 9), from highest to lowest mean content, were leucine, lysine, valine, isoleucine, phenylalanine, threonine, histidine, methionine, and tryptophan (Table 5). The most abundant non-essential amino acids (*n* = 14), from highest to lowest mean content, were glutamic acid, aspartic acid, arginine, glycine, alanine, proline, serine, tyrosine, cysteine, taurine, hydroxyproline, hydroxylysine, ornithine, and lanthionine (not reported). Lanthionine was measured at 0.00 mg/100 g protein for all samples (Table 5).

Essential amino acid content varied by location and population, with particular locations and populations having higher content on average; however, we were not able to test for significant differences between groups (i.e., locations and populations).

Samples from Chimacum had the highest mean total essential amino acid, aromatic amino acid (AAA), leucine, valine, and histidine content (Table 5). Mount Vernon samples had the lowest mean total essential amino acid, leucine, lysine, sulfur amino acid (SAA), isoleucine, threonine, histidine, and tryptophan content. Samples from Quilcene had the highest mean lysine, SAA, and threonine content. Samples from Sequim (*n* = 30; breeding lines and control varieties; Table 3) had the highest mean tryptophan content, and the lowest mean AAA and valine content (Table 5).

### Essential Amino Acid Content by Seed Source

The “Controls” seed source had the highest mean total essential amino acid, AAA, valine, SAA, and tryptophan content (Table 5). Population 102 had the highest mean lysine and threonine content. Population 104 had the lowest mean AAA, leucine, valine, isoleucine, and histidine content. Population 106 had the highest mean leucine, isoleucine, and histidine content, and the lowest mean tryptophan content. Population 107 had the lowest mean total essential amino acid, lysine, SAA, and threonine content (Table 5).

### Satisfaction of Essential Amino Acid Daily Requirements

**Figure 1:**
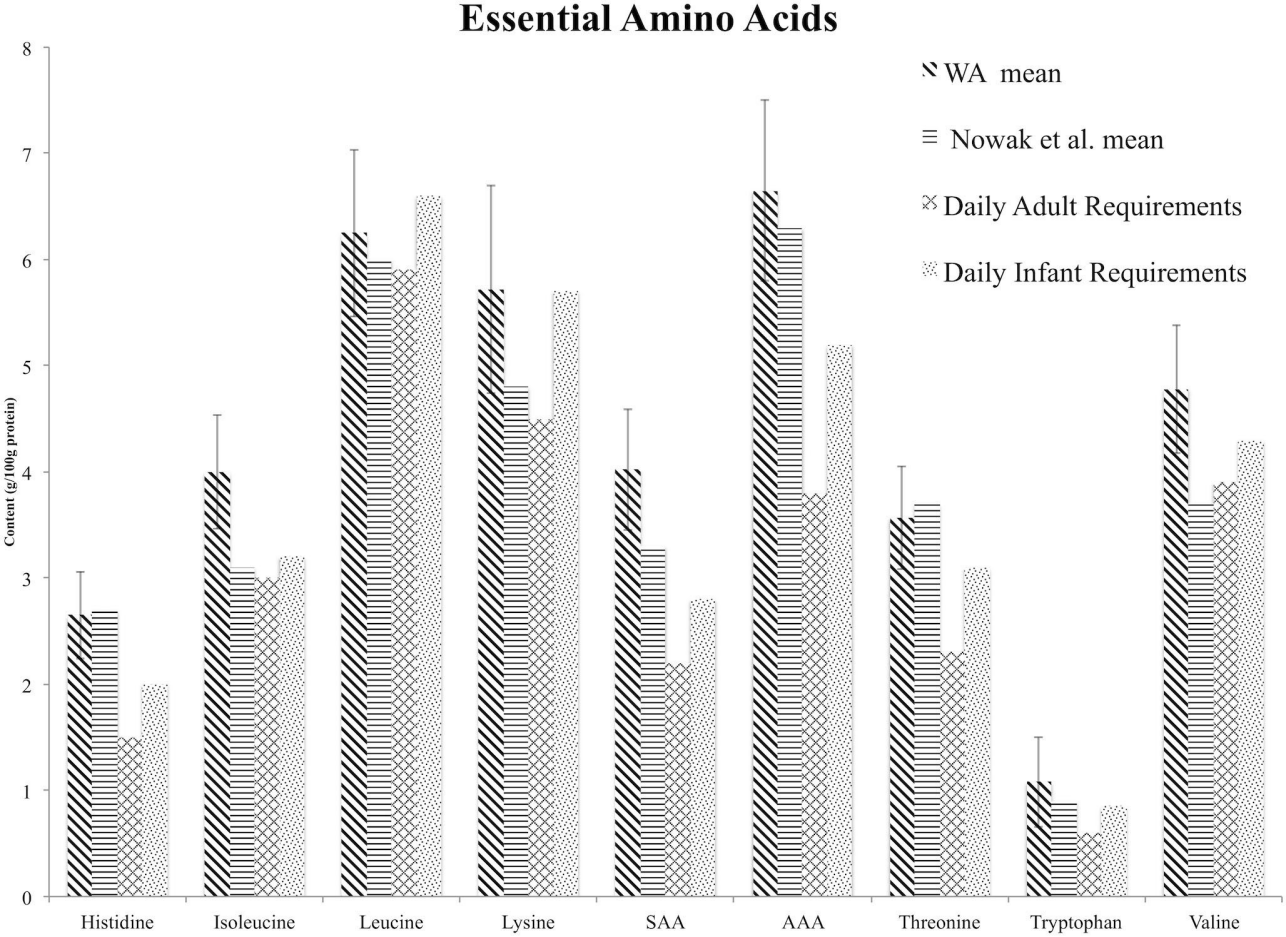
Comparison of essential amino acid mean values from the Washington grown samples (WA Mean) reported with error bars representing one standard deviation, mean values reported in the review by Nowak et al. (37), and daily adult and infant essential amino acid requirements established by the World Health Organization (52). Differences among groups are not statistically significant. SAA, sulfur amino acids; AAA, aromatic amino acids.

Mean values for histidine, isoleucine, lysine, sulfur amino acids, aromatic amino acids, threonine, tryptophan, and valine content met the daily requirements for these amino acids for all age groups (Figure 1, Table 6). The mean leucine content for locations and populations did not meet the requirements for all groups (Table 5); however, the mean value for leucine content did meet the daily requirements of the 3–10, 11–14, 15–18, >18 year-old age groups, although the infant (0.5 year) and 1–2 year-old daily requirements were not met (Tables 4, 6). Only 9% of samples met the leucine requirements for all age groups. These samples include Kaslaea (Chimacum), 102.52 (Chimacum), CO407Dave (Chimacum), 107.84 (Quilcene), 108.18 (Quilcene), 106.37 (Quilcene), 102.08 (Sequim), 102.23 (Quilcene), and 102.17 (Quilcene). Furthermore, 8% of samples failed to meet the leucine requirements for any of the age groups. These samples include 17GR (Mount Vernon; non-irrigated), 102.17 (Sequim), 102.52 (Sequim), 102.76 (Chimacum), QQ74 (Mount Vernon; non-irrigated), 104.59 (Sequim), 108.39 (Chimacum), 104.53 (Quilcene) (Table 6; Supplementary Table 1).

**Table 6:** Number of samples (*n* = 100) that fail to meet the daily requirements for each essential amino acid within each age group, and the number of samples that meet all age group requirements for each amino acid (i.e., all met).

**Number of samples that fail to meet requirements**			
**Age (years)**	**Leu**	**Lys**	**Trp**
0.5	31	42	3
1-2	35	6	3
3-10	9	0	0
11-14	8^†^	0	0
15-18	8^†^	0	0
>18	8	0	0
All met	9	52	94

*All samples meet the histidine, isoleucine, sulfur amino acid (methionine and cysteine), aromatic amino acid (phenylalanine and tyrosine), threonine, and valine requirements for all age groups*.

† *Same samples*.

Although the mean value for lysine content met the requirements of all age groups, the mean value for lysine content of certain locations and populations failed to meet daily requirements for certain age groups (Table 5). The mean value for lysine content for Chimacum, Mount Vernon, Sequim, Population 105, Population 106, and Population 107 failed to meet the requirements of infants, but met the requirements for all other age groups (Tables 4, 6). The lysine daily requirement for all age groups was met by 52 samples, while 42 samples and 6 samples failed to meet the lysine daily requirement for infants and children 1–2 years old, respectively. The tryptophan daily requirement for infants and children 1–2 years old was not met by 3 samples for each age group. Ninety-four samples met the daily requirement of tryptophan for all age groups (Table 6; Supplementary Table 1).

### Comparison to Nowak et al. (37) Mean Values

Mean values for isoleucine, leucine, lysine, sulfur amino acids, aromatic amino acids, tryptophan and valine are higher for Washington grown samples than those reported in the review by Nowak et al. (37) (Figure 1). For histidine and threonine, the Nowak et al. (37) mean value is within one standard deviation of the mean value for the Washington grown samples. However, we were not able to test for significant differences or report measures of dispersion around the population mean for the Nowak et al. (37) samples because the raw data was not available. Differences among groups were not statistically significant. The mean value reported by Nowak et al. (37) for isoleucine, leucine, and lysine does not meet the infant daily requirement, and the mean value reported for valine does not meet the adult or infant daily requirement (Figure 1).

### Samples With the Highest and Lowest Values for Nutritional Components

The five highest and five lowest samples are reported for overall rank (total content of all nutritional components), total amino acid content, total essential amino acid content, crude protein, crude fat, and ash (Table 7).

**Table 7:** The five highest and lowest ranking samples are presented for overall rank (all nutritional components combined), total amino acids (AA), total essential AA (TEAA), crude protein, crude fat, and ash.

**Rank**	**Overall**	**TAA**	**TEAA**	**Crude Protein**	**Crude Fat**	**Ash**
1	C4D•	102.17t	102.17t	104.27•	107.78^†^	3UISEΦ
2	102.52•	C4D•	102.52•	106.49^†^	106.49t	JSΦ
3	Kaslaea•	102.52•	C4D•	108.54•	106.49^†^	QQ74Φ
4	102.08^†^	Kaslaea•	Kaslaea•	104.20•	Cherry Vanilla^†^	17GRΦ
5	102.17t	102.08^†^	102.08^†^	C4D^†^	107.07t	BaerΦ
96	17GRΦ	102.76•	QQ74Φ	102.09t	JSΦ	102.52^†^
97	104.59^†^	17GRΦ	102.76•	BaerΦ	106.37^†^	106.37t
98	102.76•	102.52^†^	102.52^†^	QQ74Φ	3UISEΦ	107.07t
99	102.52^†^	QQ74Φ	102.17^†^	107.07t	107.84t	108.42^†^
100	102.17^†^	102.17^†^	17GRΦ	QQ74Φ	104.38t	104.77t

*A rank closer to zero corresponds to higher rank; rank 1 and 100 correspond to the highest and lowest ranks, respectively. Samples were either grown in 2016 in Chimacum (•), Sequim (†), Quilcene (t), or Mount Vernon (Φ). The variety CO407Dave is abbreviated as C4d and the accession Japanese Strain is abbreviated as JS*.

Samples 104.27 (Chimacum), 106.49 (Sequim), 108.54 (Chimacum), 104.20 (Chimacum), and CO407Dave (Sequim) had the highest crude protein content, while 102.09 (Quilcene), Baer (Mount Vernon), QQ74 (Mount Vernon), 107.07 (Quilcene), and QQ74 (Mount Vernon) had the lowest crude protein content.

Overall rank was highest for CO407Dave (Chimacum), 104.52 (Chimacum), Kaslaea (Chimacum), 108.08 (Sequim), and 102.17 (Quilcene) (Table 7). Samples 102.52 (Sequim) and 102.17 (Sequim) were the two lowest ranked samples overall for total content of all nutritional components. Additionally, 17GR (Mount Vernon), 104.59 (Sequim), and 102.76 (Chimacum) were among the five lowest ranked samples for total content of all nutritional components.

These same samples were similarly ranked for total amino acid content and total essential amino acid content, although the order differed slightly. Total amino acid content was highest for 102.17 (Quilcene), CO704Dave (Chimacum), 102.52 (Chimacum), Kaslaea (Chimacum), and 102.08 (Sequim) while total amino acid content was lowest for 102.76 (Chimacum), 17GR (Mount Vernon), 102.52 (Sequim), QQ74 (Mount Vernon), and 120.17 (Sequim). Total essential amino acid content was highest for 102.17 (Quilcene), 102.52 (Chimacum), CO407Dave (Chimacum), Kaslaea (Chimacum), and 102.08 (Sequim); total essential amino acid content was lowest for QQ74 (Mount Vernon), 102.76 (Chimacum), 102.52 (Sequim), 102.17 (Sequim), and 17GR (Mount Vernon).

### Correlations Between Nutritional Components

Total carbohydrate content is negatively correlated with crude protein and ash content (Supplementary Table 2). Crude protein content is positively correlated with moisture and ash content.

Total non-essential amino acid content is positively correlated with total essential amino acid content, crude protein, moisture and ash content, and negatively correlated with total carbohydrate content. Each of the non-essential amino acids are positively correlated with total essential and non-essential amino acid content, except for hydroxyproline, hydroxylysine, and ornithine content. Ash content is positively correlated with hydroxyproline, hydroxylysine, aspartic acid, glutamic acid, proline, cysteine, valine, isoleucine, leucine, phenylalanine, and histidine content (Supplementary Table 2).

Total essential amino acid content is positively correlated with crude protein, and moisture, and negatively correlated with total carbohydrate content. Each of the essential amino acids is positively correlated with total essential amino acid content and crude protein content (Supplementary Table 2).

### Principal Component Analysis

**Figure 2:**
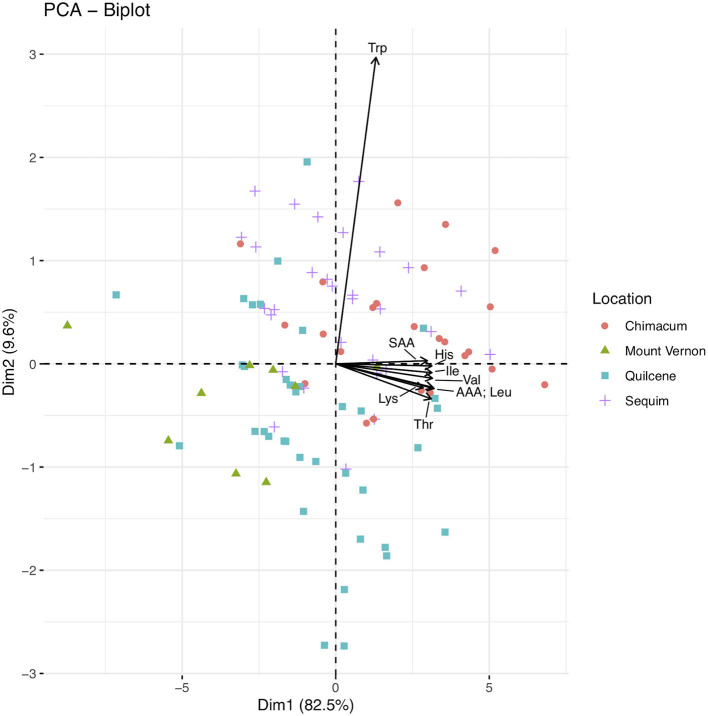
Biplot for principal component analysis (PCA) of the essential amino acid dataset (*n* = 100). Principal component one (i.e., Dim1; 82.5% of variance) and two (i.e., Dim2; 9.6% of variance) are plotted as the x- and y- axis, respectively. Essential amino acids are abbreviated using standard three letter abbreviations. The sulfur and amino acids (methionine and cysteine) aromatic amino acids (phenylalanine and tyrosine) are abbreviated as SAA and AAA, respectively. Points are color and shape coded by locations as shown in the figure legend.

Principal component analysis (PCA) of essential amino acid data identified nine principal components, with the first two principal components explaining 92.1% of the cumulative variance (Figure 2; Supplementary Table 3). Threonine, valine, isoleucine, leucine, lysine, histidine, the SAA, and the AAA have large negative loadings on principal component one, and tryptophan has a large negative loading on principal component two (Figure 2; Supplementary Table 4). The Chimacum and Sequim samples appear to cluster near the loadings, in quadrats two and three. The Quilcene and Mount Vernon samples appear to cluster on the opposite side of the biplot, in quadrats one and two.

A biplot of seed composition components further illustrates results reported in Table 5; the Mount Vernon samples have lower total essential amino acid content and lower content of each of the essential amino acids compared to samples from the other locations. Furthermore, the Mount Vernon samples cluster separately on the biplot of seed composition components (Supplementary Figure 1).

## Discussion

The quinoa germplasm pool adapted to cultivation in Washington is extremely narrow, and is primarily limited by day-length sensitivity (i.e., photoperiod sensitivity) (59). Therefore, the samples in this study represent a fraction of the over 16,000 quinoa accessions maintained in *in situ* and *ex situ* collections (6). The limited amount of germplasm adapted to Washington agroecosystems is reflected by the shared parents within the pedigrees of the breeding line populations, the small number of accessions selected and grown at Mount Vernon, and the commercial varieties included as controls at Chimacum, Quilcene, and Sequim (Table 3). However, analysis of this germplasm pool greatly enhances the data available for amino acid content in quinoa below the species level. Moreover, these samples provide a baseline for the nutritional quality of Washington grown quinoa, especially with respect to identification of limiting amino acids when compared to human health requirements. Prior to this study, little information was available regarding seed composition components and amino acid content of quinoa grown in North America. This information should inform breeding efforts and strategies, research objectives, and crop production as they relate to understanding and enhancing the nutritional quality of quinoa.

### Ash Content

Analysis of proximate content revealed considerable variation in ash content. Mean ash values were remarkably higher for samples grown at the Mount Vernon location in 2017 (4.25/100 g sample) compared to the other locations, and the mean value calculated for all samples (3.11/100 g sample) (Table 5). This may be due to the influence of genotypes (i.e., commercially available varieties), site-specific environmental characteristics, or possibly year effects (i.e., 2017 compared to 2016). Overall, mean ash content for the samples included in this study is comparable to the value reported by Navruz-Varli and Sanlier (36) (3.4%). In the review by Nowak et al. (37), the authors report ash content between 2.0 and 7.7/100 g sample, with a mean value of 3.3/100 g sample. They suggest that differences are likely due to the interactions of several factors (e.g., varieties/cultivars, analytical methods, and environmental conditions). Reguera et al. (60) observed varying concentrations of minerals in quinoa varieties grown in three different agroecological environments and hypothesize that soil composition is responsible for these differences. Similar results supporting this hypothesis are reported by Miranda et al. (61) and Prado et al. (62).

### Relationships Between Nutritional Components

Strong positive correlations exist between each essential amino acid and crude protein content. These results are supported by Gonzalez et al. (63), where they report positive correlations between amino acid content and protein content. Correlation coefficients for leucine, phenylalanine, isoleucine, tyrosine, valine, and tryptophan were significantly higher for the Bolivia/Argentina site compared to the Northwest Argentina site, while the correlation coefficients for lysine, methionine, and threonine did not significantly differ. The authors interpret these results as evidence of better adaptation of the cultivars to the Andean highland environment, because of relatively higher correlation coefficients and no significant difference in protein content found between the two agroecological regions. For researchers that do not have the capability to characterize the amino acid profile, measuring crude protein content and relying on the fact that an increase in protein content corresponds to a linear increase in essential amino acid content may suffice; however, Gonzalez et al. (63) claim that their results could indicate that the essential amino acid composition is independent of the amount of protein in the seed.

### Factors That Influence Quinoa Nutritional Protein Quantity and Quality

It should be noted that the quinoa seeds in this study were not processed prior to analysis. Processing can impact protein content, and typically consists of washing, polishing, or pearling to remove bitter saponins from the seeds before consumption. For example, Stikic et al. (64) manually dehulled seeds using a mortar to remove the pericarp and a sieve to separate the hulls, and then washed the dehulled seeds until “purified” (i.e., no foaming in rinse water from rubbing and washing seeds to remove saponins). They report a mean protein content of 17.41% for whole quinoa seeds, 15.69% for dehulled seeds, and 15.16% for purified seeds. Furthermore, they report a reduction in ash content following dehulling and sieving, from 7.06 to 3.59%, and an additional reduction to 2.24% following washing and rubbing (i.e., “purified”). Aluwi et al. (15) report significantly lower protein content for degermed Bolivian Royal, and higher protein content for scarified Cherry Vanilla. Based on the literature, the protein content reported in this study would change depending on the processing method performed on the raw quinoa before nutritional analysis.

Quinoa is often reported as having high protein content (35, 60, 64); however, quinoa protein content can be highly variable, and is often comparable to most cereals (37, 40, 65–67). Across all samples, mean protein content was 11.77/100 g sample, and ranged from 10.04 to 13.68/100 g sample (Table 5). This is comparable to values reported by Nowak et al. (37) (range = 9.1–15.7/100 g edible portion; mean = 13.1/100 g edible portion), Gonzalez et al. (63) (9.2%-15.4%), Dini et al. (68) (12.5%) and Miranda et al. (69) (11.3–16.1%), and lower than values reported by Reguera et al. (60) (14.8–17.5%), Vidueiros et al. (70) (14.5–18.2%), Koziol (67) (16.5%), Wright et al. (71) (16.7%), Bruin (72) (15.6%), Bhargava et al. (73) (12.5–21.0%), and Mota et al. (74) (12.2-16.3%). Rojas et al. (6) provide a summary of the nutritional value of a germplasm collection maintained in Bolivia, and report protein content from 10.21 to 18.39%.

In quinoa, the effects of genotype, environment, management practices, and their interactions (G × E; G × E × M) on nutritional parameters have not been completely elucidated. Protein quantity is influenced by factors such as soil fertility, environment, and genotype (6). Several studies support the hypothesis that environmental and agroecological conditions can influence quinoa protein content. In a study of three quinoa cultivars (Regalona, Salvedo, Titicaca) grown in three different agroecological zones (Spain, Peru, Chile), Reguera et al. (60) found no differences in protein content among the varieties within a location; however, protein content significantly differed between locations. Präger et al. (75) show that environmental conditions can modulate protein content in a genotype dependent manner. For example, the cultivars Jessie and Titicaca showed significant differences across years for protein content, while protein content values for Zeno and Puno remained stable across the 2 years at the one location studied. To this point, results from Gonzalez et al. (63) showed both significant inter- and intra-cultivar differences in protein content, and no differences for particular cultivars, although minimum, maximum, and mean values for both sites were practically the same in a study of 10 cultivars grown at two different agroecological sites. The authors suggest that complex underlying environmental and/or G × E interactions contribute to the significant changes in protein content in quinoa seeds from different agroecological regions for certain genotypes, but not others. Miranda et al. (61) found no difference in protein content values in a study of two cultivars (Regalona and Villarica) grown in two contrasting environments in Chile, perhaps because of broad adaptability to contrasting environmental conditions. Genotype dependent susceptibility to the effect of various factors (e.g., agro-environmental conditions) on nutritional protein quality should be further investigated and considered in the context of germplasm expansion to novel regions outside the environment the germplasm was developed in.

This study did not explicitly test for the influence of G × E interactions on quinoa nutritional parameters. However, our results provide insight into possible G × E interactions and their influence on Washington grown quinoa essential amino acid content. **Two** breeding lines in particular (102.17-Quilcene and 102.52-Chimacum) ranked in the top five among all samples in terms of overall content of nutritional parameters, total amino acid content, and essential amino acid content; however, these same two breeding lines ranked in the bottom five among all samples when grown at the Sequim location (Table 7). Soil analysis of the field locations (Table 1) reveals distinct differences with respect to environmental (e.g., annual precipitation) and soil quality characteristics that may be contributing to variation in the content of nutritional attributes. The Sequim location can be characterized by lower annual precipitation, although the farmer-collaborator did irrigate the field. Furthermore, the Sequim location had the lowest soil nitrate levels (3.8 mg kg^−1^) compared to Quilcene (16.6 mg kg^−1^) and Chimacum (21.5 mg kg^−1^). It is possible that G × E interactions contributed to the stark differences in nutritional quality observed among replicated genotypes, and that perhaps soil nitrate levels are a driver of these interactions.

Gomaa (76) found that increased nitrogen application increased quinoa protein content, and Gonzalez et al. (63) showed that protein content and essential amino acid content are positively correlated in quinoa. We expect soil nitrate levels to influence protein content, and consequently essential amino acid content, but were not able to adequately test this with the present study. The Chimacum location had the highest soil nitrate levels, and samples grown at this location had the highest mean protein content (12.25/100 g sample), highest total amino acid content (10.82/100 g crude protein), and highest total essential amino acid content (34.53/100 g crude protein), albeit within one standard deviation of the mean values for the other locations (Table 5). One sample from Sequim (102.08) ranked fourth overall in terms on nutritional composition, fifth for total amino acid content, and fifth for essential amino acid content. This sample belongs to Population 102, which had two other breeding lines ranked among the top five samples for the aforementioned traits. This population has CO407Dave as the female parent; a sample of CO407Dave from Chimacum was ranked number one overall, second for total amino acid content, third for total essential amino acid content, and CO407Dave from Sequim is ranked fifth overall for crude protein content (Table 7). It's possible that these specific breeding lines are capable of efficient nitrogen uptake for protein and amino acid synthesis, regardless of the location, perhaps because of the presence of CO407Dave in their pedigree. A lack of sufficient replication prohibits us from explicitly testing this hypothesis and making a definite conclusion. Studies are currently underway to better understand these relationships.

Perhaps more important than protein content is protein quality, or the composition with regards to the proportion of essential amino acid content (35). Quinoa is often regarded as having high lysine, leucine and sulfur amino acid content, especially in comparison to cereal crops (35, 38, 77–79). Leucine, lysine, and valine had the highest mean values, which is a finding supported by previous studies (37, 74, 75). For the non-essential amino acids, glutamic acid, aspartic acid, and arginine were the most abundant, which is also supported by other studies (64, 69, 75). Total essential amino acid content ranged from 30.78 to 37.32/100 g crude protein, which is similar to the total essential amino acid values reported by Miranda et al. (69) (34.1–35.9/100 g crude protein). Our values fall within a narrower range than those reported by Gonzalez et al. (63) (8.0–37.5/100 g crude protein), and are higher than the values reported by Präger et al. (75) (20.4–30.0/100 g) (Supplementary Table 5).

The aforementioned factors (e.g., genotype, environment, management, and their interactions) that can influence quinoa protein quantity also have the potential to influence quinoa protein quality (i.e., amino acid content and composition) (6). However, the primary factors responsible for influencing amino acid content in quinoa are not definitively agreed upon in the literature. For example, Miranda et al. (69) provide evidence in support of Wright et al. (71) and claim that the genetic characteristic of the quinoa genotypes decisively influences amino acid content. In a study of three Chilean landraces exposed to two levels of salinity under controlled conditions, Aloisi et al. (80) present results that support strong genotype-dependent responses to salinity with respect to essential amino acid content. Conversely, Gonzalez et al. (63) propose that amino acid content is higher among germplasm when grown in the environment to which it is adapted, based on the idea that cultivars growing in the geographic area of origin would exhibit better gene expression related to amino acid synthesis. Moreover, they state that both environmental and climatic factors influence the amino acid composition of quinoa cultivars growing in different agroecological zones. They report significant differences in total amino acid content and total essential amino acid content between the two agroecological sites examined for nine out of ten cultivars. The findings of Reguera et al. (60) and Präger et al. (75) support Gonzalez et al. (63) and the case for complex G × E interactions influencing essential amino acid content, as both studies found considerable variation in essential amino acid content depending on the variety and area of cultivation.

### Identification of Limiting Amino Acids

Quinoa is often referred to as a “complete protein” because it contains all the essential amino acids; however, it is better described as “nearly complete” because of limiting amino acid content. Our study provides data on essential amino acid profiles for 100 distinct samples, representing 92 unique commercial varieties, landraces, and advanced breeding lines adapted to cultivation in Washington State, and evaluates the nutritional protein quality of each sample compared to the requirements of all age groups (52). We identify samples that fail to meet the daily requirements of all age groups for leucine, lysine, and tryptophan. Of the samples analyzed, only nine met the leucine requirements for all age groups. These samples include 7 advanced breeding lines (102.52, 107.84, 108.18. 106.37, 102.08, 102.23, and 102.17) and two commercial varieties (Kaslaea, CO407Dave); five samples were grown at Quilcene, three samples were grown at Chimacum, and one sample was grown at Sequim. Moreover, 52 samples and 94 samples met the lysine and tryptophan requirements for all age groups (Supplementary Table 1). These results provide the first report of leucine as a limiting amino acid in quinoa.

Quinoa protein quality is often compared to that of casein, a milk protein, because of similar values for protein digestibility and essential amino acid content (33, 67, 81, 82). In a joint report, the FAO/WHO/UNU Expert Consultation on Protein Quality Evaluation recommended the use of the protein digestibility-corrected amino acid score (PDCAAS) method for evaluating protein quality. Casein PDCAAS values are typically close to, or exceeding, the 1.0 truncation value (83). Reports of quinoa PDCAAS values and protein digestibility are sparse in the literature; however, 82 report recalculated PDCAAS values of 0.85 (raw quinoa) and 1.00 (washed quinoa) for the 1–2 year-old age group, and 0.89 (raw) and 1.09 (washed) for the 3–10 year age group based on data from Ruales and Nair (41). Quinoa protein digestibility varies depending on genotype, processing, and evaluation method, although saponin removal and cooking generally improves digestibility (34, 65, 81, 84–87). We calculated PDCAAS values using an apparent protein digestibility value of 84.3% based on fecal protein losses in rats as reported by Ranhotra et al. (82) for the variety “Colorado D407.” Values ranged from 0.74 to 0.90 and 0.78 to 0.95 for the 1–2 and 3–10 year-old age groups, respectively (Supplementary Tables 6, 7).

The vast majority of studies that report quinoa essential amino acid content, and compare these values to daily human health requirements, either make comparisons to outdated daily requirements, or only consider requirements of the adult age group. For example, Ruales and Nair (41) report the aromatic amino acids, threonine, and lysine as the first, second, and third limiting amino acids, respectively, while Boye et al. (83) identified valine and lysine, and lysine as limited amino acids for the 1–2 and 3–10 year-old age groups using values from Ruales and Nair (41) and current WHO/UNU/FAO (52) requirements. Mahoney et al. (81) analyzed a single quinoa variety (Sajama; Patacamaya Agricultural Experiment Station, Lapaz, Bolivia) and identified methionine as the first limiting amino acid, followed by tryptophan. Gonzalez et al. (63) report lysine, threonine, and methionine content in relatively adequate amounts for both human and animal feeding. They also claim that content for the aromatic amino acids, isoleucine, threonine, and valine were sufficient for 10–12 year-old children. The authors identify lysine, tyrosine, and tryptophan as limiting amino acids for 2–5 year-old children. In addition to lysine and tryptophan being limiting amino acids for 2–5 year-old children, leucine is also a limiting acid for this age group for our samples. The comparisons made be Gonzalez et al. (63) are made to the FAO/WHO guidelines published in 1990, even though updated guidelines were published in 2007. Repo-Carrasco et al. (88) cite studies conducted at the Universidad Nacional Agraria La Molina (UNALM) that found that one cultivar of quinoa, Amarilla de Marangani, does not have any limiting amino acids, although no information is provided concerning the age group or requirements that form the basis for this comparison.

The potential for quinoa to adapt to diverse agroecological conditions and contribute to food and nutritional security is generating worldwide interest and contributing to the current global expansion of quinoa. Recent studies have evaluated the potential of quinoa in novel production environments, with a focus on agronomic traits and nutritional quality, especially essential amino acid content. Indirect evaluation of quinoa nutritional protein quality, for studies that do not make a direct comparison, can be accomplished by comparing published values for essential amino acid content to daily essential amino acid requirements. For example, the mean value of the four cultivars (Zeno, Jessie, Puno, Titicaca), and each individual cultivar, reported by Präger et al. (75) for a single location and 2 years, fail to meet leucine, lysine, and histidine requirements for all age groups for the first year of their study, while only one cultivar (Puno) met the requirements for isoleucine, the sulfur and aromatic amino acids, and threonine (Supplementary Table 5). In the second year of the study, the mean value of the four cultivars, and each individual cultivar, failed to meet the leucine, lysine, sulfur and aromatic amino acid requirements; Puno met the isoleucine requirement within one standard error of the mean, although the mean value was lower than the requirement. All cultivars and the mean value for all four cultivars met the tryptophan requirements for both years (Supplementary Table 5). The amino acid values reported by Miranda et al. (69) for six genotypes and one genotype failed to meet the lysine and leucine requirements for all age groups, respectively. The ability of quinoa to meet global health challenges depends in part on the ability to meet essential amino acid requirements for all age groups.

## Conclusions

This study provides a baseline analysis of the nutritional quality of quinoa grown in Washington state. For the germplasm tested, protein content is lower than values reported in the literature; however, essential amino acid content is generally higher. Mean essential amino acid values meet the daily requirements for infants and adults, except for the amount of leucine required by infants. This is the first report of leucine as a limiting quinoa amino acid. We identify 9% of the samples that meet the leucine requirements for all age groups, and 8% of the samples that fail to meet the requirements for any age group. Lysine requirements for the <0.5 and 1–2 year-old age groups were not met by 31 and 35 genotypes, respectively. Tryptophan requirements for the <0.5 and 1–2 year-old age groups were each not met by three genotypes. This study greatly augments the amount of data available for quinoa nutritional quality below the species level, and provides the first in-depth report of the protein quality of quinoa grown in North America. The information reported in this study will be useful for guiding research objectives and breeding strategies, in pursuit of supporting the global expansion of quinoa and the potential for quinoa to contribute to addressing global public health challenges. Effective breeding strategies for improving quinoa protein quality should focus on identifying limiting amino acids, the factors that influence amino acid content, and increasing the content of limiting amino acids to improve PDCAAS. Moreover, increased lysine and sulfur amino acid content are important targets, because these amino acids are limiting in most common cereals (e.g., wheat and maize), in addition to leucine content. Future work must be context specific with respect to germplasm adapted to the target production environment and culture.

## Data Availability Statement

All datasets presented in this study are included in the article/Supplementary Material. The raw data supporting the conclusions of this manuscript will be made available by the authors, without undue reservation, to any qualified researcher.

## Author Contributions

KM conceived the project and provided edits to the manuscript. EC performed data collection and analysis and wrote the manuscript. All authors contributed to the article and approved the submitted version.

## Conflict of Interest

The authors declare that the research was conducted in the absence of any commercial or financial relationships that could be construed as a potential conflict of interest.
