# Corrigendum: Seed Composition and Amino Acid Profiles for Quinoa Grown in Washington State

**DOI:** 10.3389/fnut.2020.605674

**Published:** 2020-11-09

**Authors:** Evan B. Craine, Kevin M. Murphy

**Affiliations:** Department of Crop and Soil Sciences, Washington State University, Pullman, WA, United States

**Keywords:** quinoa, essential amino acid, limiting, complete protein, protein quality

In the original article, there was a mistake in the legend for **Table 1** as published: important citations were not included for the referenced studies. The correct legend appears below.

**Table 1** **∣** **Site characteristics for each year and location that samples were randomly selected from for chemical analysis. Raw quinoa seed sent for analysis was grown in 2016 and 2017 in western Washington as part of two separate experimental designs** **(****13**, **49****)**.

In the original article ***(Hinojosa et al., 2019; Kellogg and Murphy*** ***2019)***was not cited in the article. The citation has now been inserted in ^**^Methods Section^**^, ^**^Study Region and Field Trials^**^, ^**^Paragraph one^**^ and should read:

^**^Raw quinoa seed sent for analysis was grown in 2016 and 2017 in western Washington as part of two separate experimental designs **(****13**, **49****). Site characteristics for all locations are summarized in Table 1. In 2016, F5:F6 advanced breeding lines and control varieties were planted on three organic farms in Chimacum (Finnriver Farm; 48°0'29“N 122°46'12”W), Quilcene (Dharma Ridge Organic Farm; 47°55'04.0“N 122°53'23.2”W) and Sequim (Nash's Organic Produce; 48°08'31“N 123°07'19”W) on the Olympic Peninsula. Control varieties included Cherry Vanilla (Wild Garden Seed, Philomath, OR, US), CO407Dave (PI 596293, USDA Plant Introduction, Ames, Iowa) and Kaslaea (Ames 13745, USDA Plant Introduction, Ames, Iowa). At each location, advanced breeding lines and control varieties were planted in single hand-sown plots that measured 4.9 m in length and 40.64 cm from center and were seeded at a rate of 4 g row m**^**−1**^ **in an augmented randomized complete block design (ARCBD). An ARCBD uses control varieties to account for field variation by replicating control varieties across blocks; control varieties can be used as covariates to make spatial adjustments across blocks. This design is useful for evaluating advanced breeding lines when seed quantity is low, land and other resources are limited, and when many advanced breeding lines must be evaluated**.^**^

The authors apologize for this error and state that this does not change the scientific conclusions of the article in any way. The original article has been updated.
