# Supplementary figures and images for: Seed Composition and Amino Acid Profiles for Quinoa Grown in Washington State
Source: Front Nutr. 2020 Aug 12;7:126. doi: 10.3389/fnut.2020.00126 (PMC7434868; doi:10.3389/fnut.2020.00126)

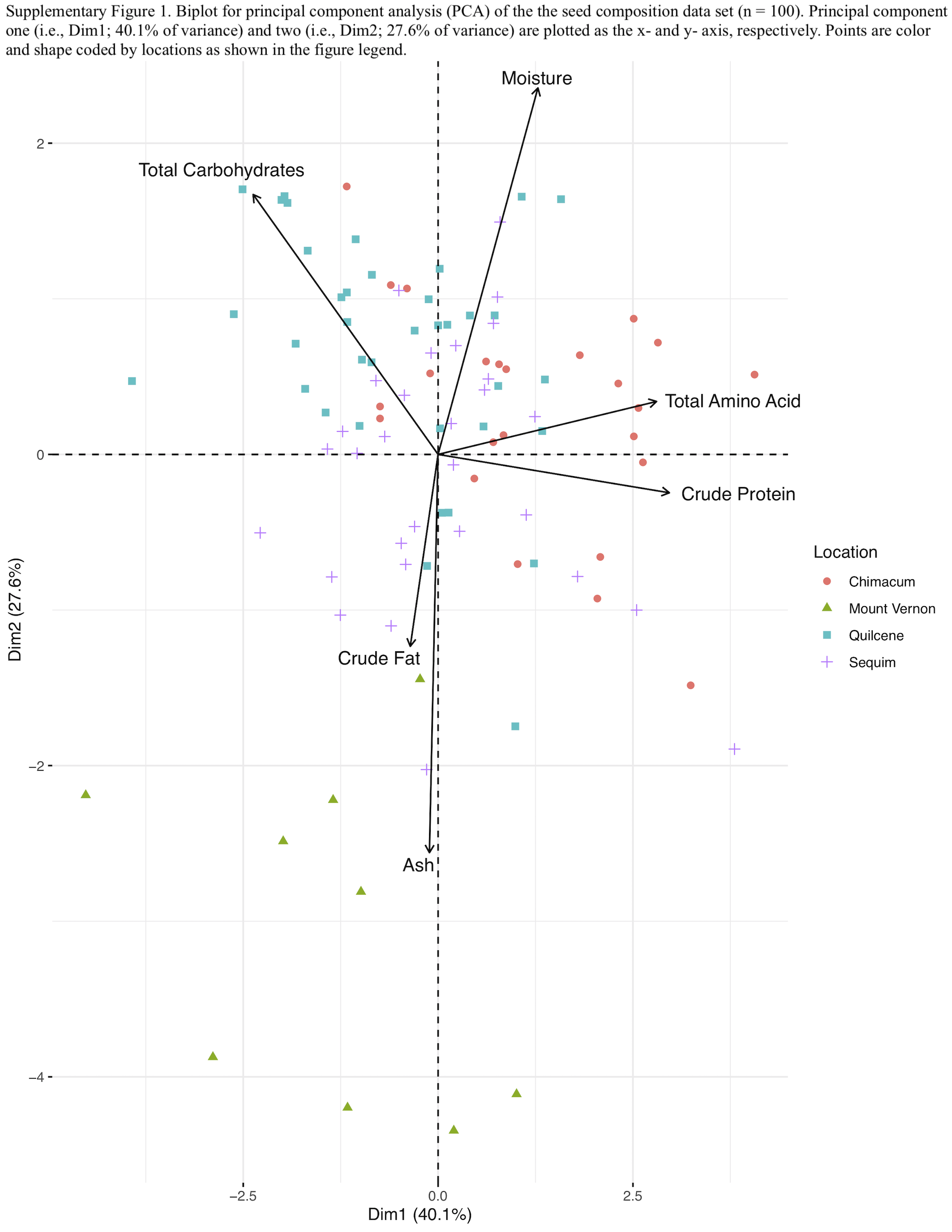

Supplement: Supplementary file 8 [file Image_1.tiff]
